# Ion Channels in the Immune Response of Asthma

**DOI:** 10.70322/jrbtm.2024.10019

**Published:** 2024-11-15

**Authors:** Liang Yan, Lu Zhang, Kenneth Ogunniyi, Liang Hong

**Affiliations:** 1 Department of Medicine, University of Illinois at Chicago, Chicago, IL 60612, USA; 2 Department of Physiology and Biophysics, University of Illinois at Chicago, Chicago, IL 60612, USA; 3 Department of Biomedical Engineering, University of Illinois at Chicago, Chicago, IL 60612, USA

**Keywords:** Asthma, Lung disease, Ion channel, Immune cells, Inflammation

## Abstract

Asthma is a common respiratory disorder characterized by chronic inflammation of the lower airways, contributing to significant morbidity, mortality, and a substantial global economic burden. It is now understood as a heterogeneous condition, with ongoing research shedding light on its complex immunological underpinnings. Ion channels, which are specialized transmembrane proteins that facilitate ion movement based on electrochemical gradients, play a crucial role in the pathophysiology of asthma. Ion channels regulate essential processes like maintaining epithelial hydroelectrolyte balance and also play a role in modulating immune responses involved in asthma. We discuss the connection between ion channel activity and immune regulation in asthma, focusing on ion channel regulation of immune cell behavior, airway hyperresponsiveness, and inflammation in asthma. Understanding ion channels in asthma could lead to the development of targeted therapies modulating their activity, thereby enhancing disease management and patient outcomes.

## 1. Introduction

Asthma is a major global health issue, impacting around 262 million people worldwide and contributing to an estimated 455,000 deaths each year [1,2]. Over the past decade, the prevalence of asthma has steadily risen, placing an increasing strain on healthcare systems globally [3,4]. Despite advances in our understanding of the disease, asthma remains a chronic and potentially life-threatening condition, requiring ongoing management and treatment.

Research has shown that asthma is a heterogeneous disorder primarily affecting the conducting airways [5,6]. It manifests in two primary phenotypes: allergic and non-allergic asthma. The allergic form generally aligns with the Type 2 inflammation category, where the immune response is driven by Th2 cells and involves eosinophilia, IgE production, and heightened reactivity to allergens triggered by environmental allergens. The non-allergic form primarily involves Th1 and Th17 cells and is characterized by non-eosinophilic, often neutrophilic inflammation triggered by irritants or infections can also involve elements of Type 2 (type 2-low) inflammation. Non-allergic asthma is induced by infections, irritants, stress, and obesity, primarily activating innate immunity. Both phenotypes share common features, including airway inflammation, hyperresponsiveness, and obstruction. Asthma has been associated with ion channel activities, and recent research has highlighted the role of ion channels in asthmatic lung inflammation [7,8].

Ion channels are specialized transmembrane proteins that regulate the flow of ions, such as calcium (Ca^2+^), potassium (K^+^), sodium (Na^+^), chloride (Cl^−^), and proton (H^+^) channels across the cell membrane [9]. Ion channel dysfunction has been implicated in human diseases [10–12]. This ion movement is critical for maintaining cellular homeostasis and enabling various cellular functions, including muscle contraction, immune cell activation, and epithelial barrier integrity. By influencing the behavior of immune cells, such as mast cells, eosinophils, and T cells, ion channels contribute to the complex interplay between airway inflammation and immune regulation. Understanding the role of ion channels in the immune processes that drive asthma is essential for the development of new therapeutic strategies to protect against disease exacerbations. This review provides an updated exploration of the role of immune cell ion channels in asthma pathogenesis, focusing on their contribution to immune regulation and airway inflammation. By delving into recent advances in ion channel research, we aim to illuminate how modulating ion channel activity could provide novel therapeutic avenues for improving asthma management and patient outcomes.

## 2. Immune Regulation in Asthma Pathogenesis

**Figure 1.**
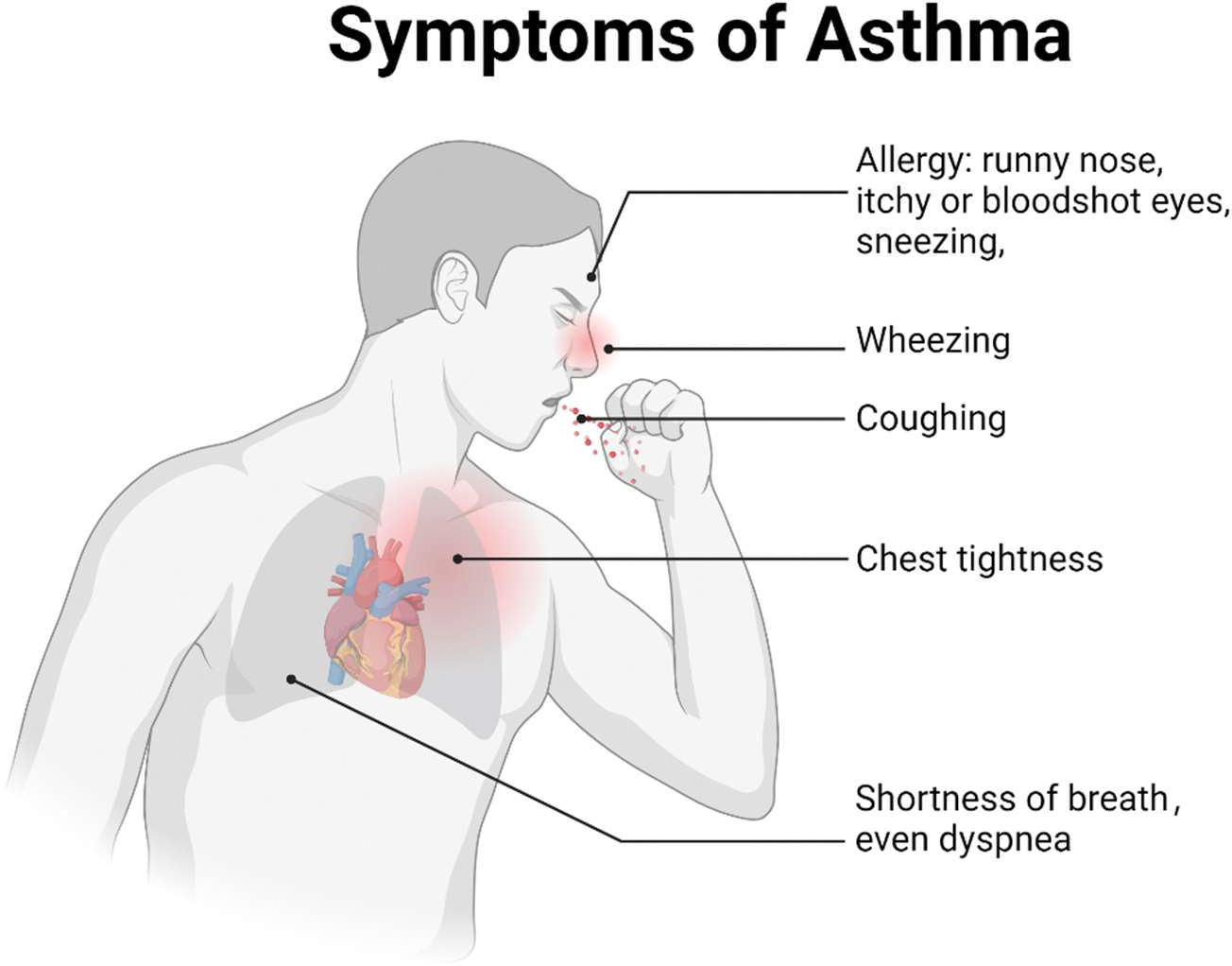
Symptoms of asthma. Key symptoms include wheezing, shortness of breath, coughing, chest tightness, and dyspnea. Allergic asthma is usually triggered by environmental allergens and is commonly associated with allergy symptoms such as a runny nose, sneezing, and itchy or red eyes. Non-allergic asthma is typically triggered by factors such as cold air, smoke, pollution, or respiratory infections. Non-allergic asthma is often associated with respiratory irritants rather than allergens.

Asthma is a chronic inflammatory disorder of the airways triggered by various environmental factors, such as allergens, infections, and tobacco smoke. It is characterized by bronchial hyperresponsiveness, airway remodeling, narrowing of the airway lumen, and excessive mucus production [8] (Figure 1). The pathophysiology of asthma is driven by complex interactions between various cell types, including resident cells like epithelial and smooth muscle cells, as well as circulating immune cells [13]. The immune system plays a central role in both allergic and non-allergic asthma. In allergic asthma, immune responses are typically initiated by environmental allergens that activate adaptive immune pathways. In contrast, non-allergic asthma often involves innate immune responses triggered by irritants, infections, or stress. In both forms of asthma, immune cells such as T cells, eosinophils, and mast cells contribute to airway inflammation, which drives the hallmark features of the disease, including airway obstruction and hyperreactivity.

### 2.1. Allergic Asthma

**Figure 2.**
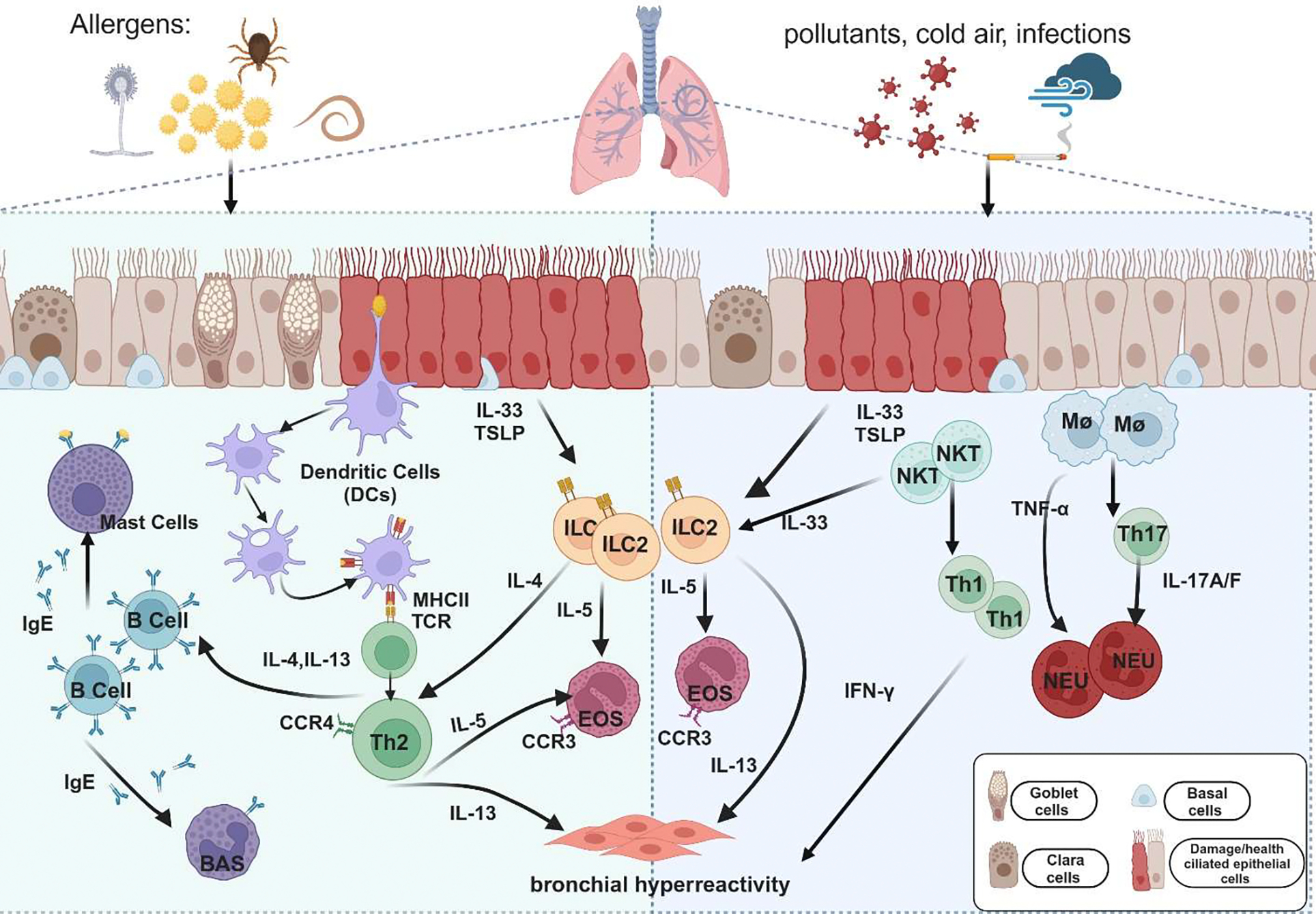
Cellular pathways and cytokines are involved in the immune mechanisms of allergic and non-allergic asthma. Upon exposure to allergens (e.g., pollen, dust mites, mold, and helminths) or non-allergic triggers (e.g., cigarette smoke, cold air, and pollution), epithelial cells secrete IL-33 and thymic stromal lymphopoietin (TSLP), which activate immune cells such as dendritic cells (DCs), macrophages (Mφ), and natural killer T (NKT) cells. In allergic asthma (Left panel), DCs capture and present antigens via major histocompatibility complex type II (MHC II), leading to the activation of T helper type 2 (Th2) cells. Th2 cells release cytokines, including IL-4, IL-5, and IL-13, which promote immunoglobulin E (IgE) production, eosinophil (EOS) recruitment, and airway hyperresponsiveness (AHR) in allergic asthma. Alongside Th2 cells, type 2 innate lymphoid cells (ILC2s) are activated by IL-33 and TSLP and play a crucial role in allergic inflammation. ILC2 cells secrete large amounts of IL-5 and IL-13, contributing to eosinophil recruitment and AHR, further amplifying type 2 inflammation. In non-allergic asthma (Right panel), neutrophil-dominated inflammation is driven primarily by Th1 and Th17 cells. Th1-related cytokine interferon-gamma (IFN-γ) and Th17-related cytokines IL-17A and IL-17F induce neutrophilic (NEU) lung inflammation and AHR. Tumor necrosis factor-alpha (TNF-α), produced by macrophages and mast cells, promotes neutrophil chemotaxis, intensifying the inflammatory response. Among airway cells, basal cells are essential for airway repair and regeneration, showing increased proliferation in response to inflammation or damage, particularly in allergic asthma. Goblet cells, responsible for mucus production, also become more active in allergic asthma, leading to excessive mucus and airway obstruction. This process is less pronounced in non-allergic asthma; Ciliated cells are involved in clearing mucus and trapped pathogens. Their function is often impaired in allergic asthma due to inflammation, leading to decreased mucociliary clearance; Clara cells secrete protective proteins and participate in airway repair. Their function may be reduced by inflammation in allergic asthma, whereas in non-allergic asthma, they primarily respond to physical or chemical irritants.

Allergic asthma is the most common type of asthma, usually starting in early childhood and marked by sensitivity to specific allergens [14]. Initiation of immunity often begins with antigen-presenting cells (APCs), such as dendritic cells (DCs), whose role is mainly in capturing and presenting antigen through the major histocompatibility complex type II (MHC II) molecule. This presentation triggers the activation of T helper type 2 (Th2) cells, which are central to the allergic response in asthma. Th2 cells can release cytokines, such as interleukin-4 (IL-4), interleukin-5 (IL-5), and interleukin-13 (IL-13), which are critical for the development and perpetuation of allergic inflammation [15]. IL-5 promotes the maturation and release of eosinophils from the bone marrow, while IL-13 stimulates the proliferation of B cells that produce immunoglobulin E (IgE). IL-4 is particularly important for inducing B cells to differentiate into plasma cells and switch to producing IgE [16]. Allergen-specific IgE molecules bind to effector cells like mast cells and basophils. Upon re-exposure to the allergen, these IgE molecules cross-link, triggering the degranulation of preformed mediators such as histamine and tryptase, as well as the release of additional inflammatory mediators, and lead to the clinical symptoms of asthma [17]. Additionally, IL-4 enhances the polarization of naive T cells into Th2 cells [18], thereby amplifying the overall Th2-driven inflammatory response, which is characteristic of allergic asthma. This feedback loop perpetuates immune activation and contributes to the chronic nature of asthma (Figure 2). Several cell types, including Th2 cells, eosinophils, and innate lymphoid cells, are implicated in the pathogenesis of allergic asthma.

#### 2.1.1. Th2 Cells

Asthma is traditionally identified as a Th2-driven condition, where Th2 cells activated in the respiratory tract play a key role in the immune response, promoting pulmonary eosinophilia and excessive mucus production. Unsupervised clustering algorithms have identified distinct asthma endotypes based on Th2 activity, broadly categorizing asthma into Th2-high (Th2hi) and Th2-low (Th2lo) groups. These classifications are defined by the presence or absence of Th2-associated cytokines such as IL-4, IL-5, IL-13, and eosinophils in the blood and tissues [19]. In experimental ovalbumin (OVA)-induced asthma models, genetic or antibody-mediated depletion of CD4^+^ T cells has been shown to abolish key features of asthma. Conversely, the adoptive transfer of Th2-polarized CD4^+^ T cells from mice expressing an OVA-specific T cell receptor (TCR) can induce asthma-like features, further demonstrating the pivotal role of Th2 cells in disease development [20]. Additionally, chemokine receptors such as C-C chemokine receptor 3 (CCR3), C-C chemokine receptor 4 (CCR4), C-C chemokine receptor 8 (CCR8) and C-X-C chemokine receptor 4 (CXCR4) are also key in Th2-driven asthma. CCR3 is expressed in Th2 cells, and their associated ligands play critical roles in mediating the recruitment and activity of Th2 cells in the lungs. CCR4, which regulates the chemotaxis of Th2 cells, is associated with increased levels of its ligands CCL17 and CCL22 in patients with allergic asthma. CCR8 has been linked to eosinophilia and airway hyperresponsiveness (AHR), and its expression may be elevated in Th2 cells within the lungs and airways of allergic asthmatics. CXCR4, which plays a role in the migration of Th2 cells into the lungs, has been identified as a potential therapeutic target. In allergic mouse models, inhibiting CXCR4 significantly reduces airway hyperresponsiveness (AHR) and inflammatory responses [14].

#### 2.1.2. Eosinophils

Eosinophils are key effector cells of the immune system, containing cytotoxic granules that play a crucial role in asthma pathogenesis. Upon activation, eosinophils take on a pro-inflammatory role, producing cysteinyl leukotrienes (Cys-LTs) and additional Th2 cytokines. These mediators intensify the inflammatory response and worsen the allergic cascade in asthma. These inflammatory mediators contribute to the perpetuation of airway inflammation and hyperreactivity [21]. Eosinophil-derived products, such as eosinophil-derived neurotoxin (EDN), eosinophilic peroxidase (EPO), major basic protein (MBP), and eosinophilic cationic protein (ECP), cause direct tissue damage and bronchial hyperresponsiveness. Furthermore, they activate adaptive immunity by influencing dendritic cells (DCs), thereby enhancing the immune response [22].

In addition to blood eosinophilia, tissue eosinophilia is a hallmark of allergic inflammation and asthma. Eosinophils accumulate at sites of allergic inflammation, contributing significantly to the development and persistence of bronchial asthma [23]. Their development in the bone marrow and migration to the pulmonary mucosa and interstitium are driven by eotaxin (a newly discovered C-C chemokine that preferentially attracts and activates eosinophil leukocytes). Studies have shown that the receptor of eotaxin, CCR3, is expressed at higher levels in the airways of asthmatic patients compared to healthy controls, facilitating the targeted recruitment of eosinophils to inflamed lung tissue [24].

#### 2.1.3. Type 2 Innate Lymphoid Cells

Innate lymphoid cells (ILCs) are a group of lymphoid cells that, unlike T cells (including NKT and mucosal-associated invariant T (MAIT) cells, B cells, NK cells, macrophages, and other myeloid cells, lack classical cell surface markers [25]. Three distinct types of ILCs have been identified: ILC-1, which produces interferon-γ (IFN-γ); ILC-2, which produces cytokines commonly associated with Th2 cells (IL-4, IL-5, and IL-13); and ILC-3, which produces IL-17 and/or IL-22 cells [26]. Notably, ILC-2 cells can produce key type 2 cytokines (IL-4, IL-5, IL-13) at levels 5–100 times higher per cell compared to Th2 cells, making them powerful drivers of type 2 inflammation [27]. Various studies using samples from bronchoalveolar lavage (BAL), sputum, nasal tissue, and blood, have demonstrated elevated levels of ILC-2s in asthmatic patients compared to healthy controls. These levels further decrease following effective therapeutic interventions [28–30].

Interestingly, ILC-2 cell numbers in the airways also rise after exposure to allergens during the pollen season and following infection with rhinovirus in an IL-33-dependent manner [31]. In response to epithelial injury, alarmins (such as IL-33) are released, which activate ILC-2 cells through pattern-recognition receptors. Once activated, ILC-2 cells release cytokines that drive pro-inflammatory pathways in asthma [32]. It has been identified that exposure to allergens containing proteases, such as Alternaria alternate or house dust mites, can cause epithelial damage, leading to the release of IL-33, which in turn activates ILC-2 cells [33,34]. Additionally, proteolytic damage to the epithelium can induce the production of leukotriene D4 (LTD4), which binds to the cysteine leukotriene receptor (CysLT1R), further regulating the activation and proliferation of ILC-2 cells [35]. Besides, ILC-2 cells play an integral role in the rapid inflammatory response of viral infections, which could be activated in response to viral infections affecting the airway epithelium and contribute to the frequent asthma exacerbations during viral infections [36,37].

### 2.2. Non-Allergic Asthma

Non-allergic asthma shares many immunopathological features with allergic asthma but is distinguished by unique immune pathways [38]. Unlike allergic asthma associated with eosinophilia and Th2 cytokines, non-allergic asthma is often marked by neutrophil-dominated inflammation and the involvement of other T helper cell subsets, such as Th1 and Th17, which produce cytokines like IL-17, IL-21, and IL-22 [17]. These cytokines contribute to the pathogenesis of non-allergic asthma by driving different inflammatory mechanisms (Figure 2).

Th1 is generally thought to inhibit the Th2 response, and the shift of the Th1/Th2 balance could protect against the development of allergic bronchial asthma [39]. Th1 cells and Th1-related cytokines, such as interferon-gamma (IFN-γ), play critical roles in patients with severe neutrophilic asthma. High levels of IFN-γ, coupled with reduced levels of secretory leukocyte protease inhibitor (SLPI), are found in patients with severe asthma [40]. IFN-γ in the airways can suppress SLPI, promoting airway hyperresponsiveness (AHR). Studies in mice have shown that elevated IFN-γ levels induce neutrophilic lung inflammation, emphysema, and AHR [41]. IL-17, a key cytokine in non-allergic asthma, plays an essential role in recruiting neutrophils into the lungs. Studies have shown elevated IL-17-related cytokine levels in the bronchial and nasal mucosa of patients with neutrophilic asthma [42]. Patients with late-onset and more severe forms of asthma often exhibit activation of the IL-17-mediated pathway and reduced airway reversibility [43]. In experimental asthma models driven by house dust mites (HDMs) or ozone exposure, IL-17A contributes to airway remodeling by promoting fibroblast proliferation [44] and counteracting the anti-inflammatory functions of regulatory T cells [45]. Furthermore, bronchial IL-17F expression is significantly higher in patients with fatal asthma compared to controls [42].

A major challenge in managing neutrophilic asthma is its resistance to corticosteroids, which is associated with the expression of TH17-related cytokines in the lungs [46]. There is a complex interaction between Th17-driven inflammation and tumor necrosis factor-alpha (TNF-α). Elevated levels of TNF-α have been observed in patients with severe steroid-resistant asthma [47]. TNF-α, primarily produced by macrophages and mast cells, promotes neutrophil chemotaxis [48]. Inhaled recombinant TNF-α administered to healthy individuals has been shown to induce AHR and airway neutrophilia [49]. AHR may result from the direct effect of TNF-α on the airway smooth muscle or indirectly the release of cysteinyl leukotrienes C4 and D4 [50]. However, in experimental models where Th17 OVA-specific T cells were adoptively transferred, neutralizing TNF-α reduced neutrophil recruitment to the lungs and improved lung function parameters, such as compliance, but had minimal impact on airway hyperresponsiveness (AHR) [43]. The complex interplay between neutrophilic inflammation and steroid resistance highlights the distinct immune profile in non-allergic asthma.

## 3. Ion Channels to Immune Response in Asthma

**Figure 3.**
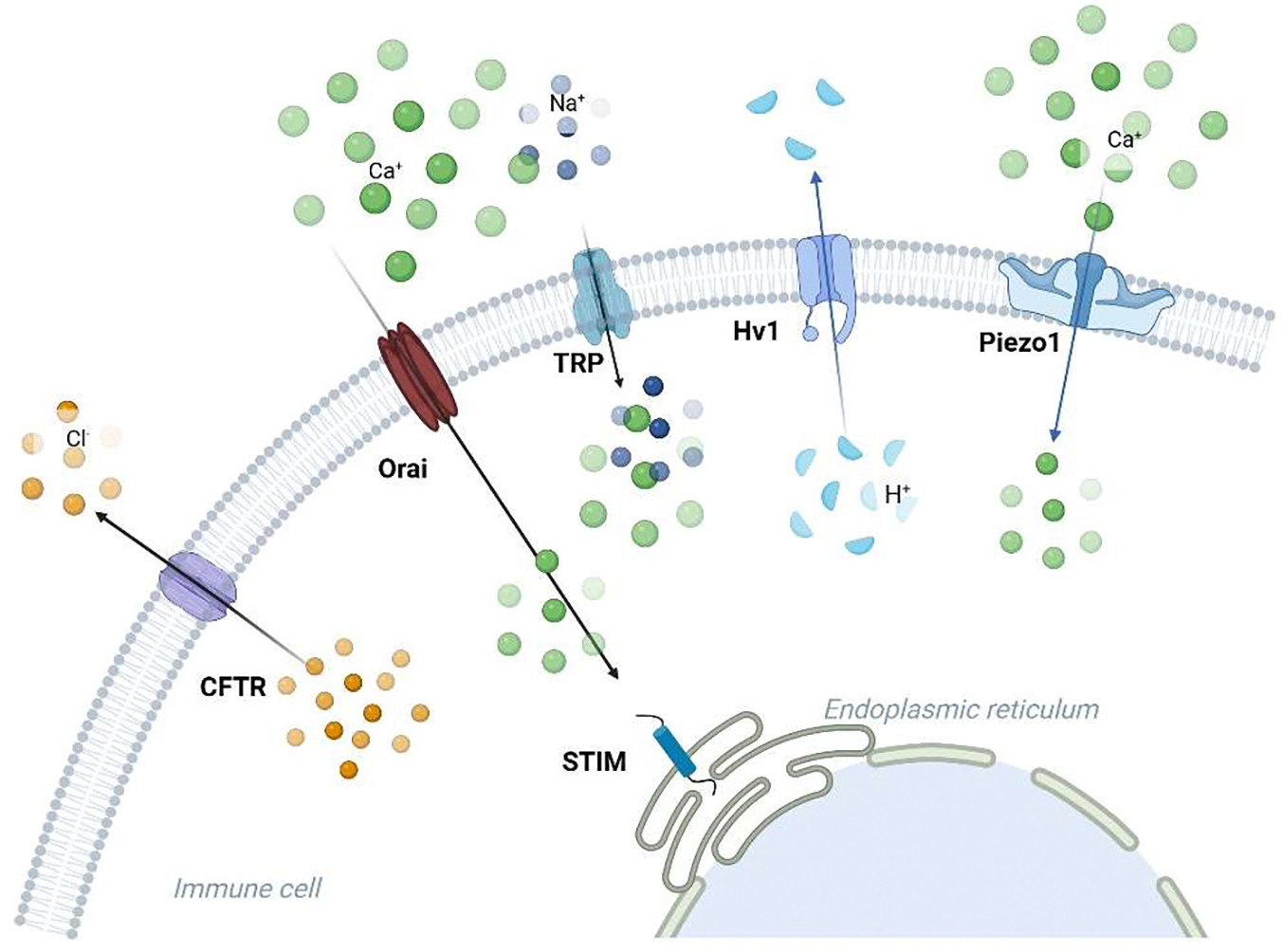
Ion channels linked to the immune response in asthma. Ca^2+^, K^+^, Cl^−^, H^+^, TRP, and Piezo1 channels are involved in asthma. Orai facilitates the entry of calcium ions (Ca^2+^), and the STIM protein on the endoplasmic reticulum membrane interacts with Orai to modulate calcium signaling in immune cells. TRP channels (such as TRPA1, TRPV1, and TRPV4) transport Ca^2+^ and sodium (Na^+^) ions, contributing to inflammation and hypersensitivity reactions. Piezo1 responds to mechanical stress, allowing calcium influx. Hv1 mediates proton (H^+^) extrusion, regulating intracellular pH and immune cell signaling. CFTR mainly manages the movement of chloride ions (Cl^−^), which is critical for maintaining ionic balance during immune activation.

While excitable cells such as muscle and nerves are well-known for firing action potentials through ion channels, non-excitable cells like leukocytes, fibroblasts, and epithelial cells also express various ion channels. Various classes of ion channels have been identified in pulmonary tissues, each playing a crucial role in regulating essential cellular functions [9]. These ion channels are essential not only for maintaining normal lung function but also for contributing significantly to the immune responses involved in asthma pathophysiology [7,8]. Ion channels are central to the inflammatory processes that characterize asthma by regulating cellular activities such as immune cell activation, secretion, and migration (Figure 3).

### 3.1. Calcium Channels

Calcium (Ca^2+^) channels play a key role in regulating immune cell function, particularly through controlling cytosolic Ca^2+^ signals. These signals are essential for immediate immune responses, such as mast cell degranulation, and long-term processes like T cell proliferation and cytokine production [51]. One of the major mechanisms for Ca^2+^ entry in immune cells is store-operated Ca^2+^ entry (SOCE), which is triggered by the depletion of Ca^2+^ stores in the endoplasmic reticulum (ER). A key component of SOCE is the calcium release-activated calcium current (CRAC), which is specifically mediated by the ER Ca^2+^ sensor stromal interacting molecules (STIM) and the plasma membrane pore-forming proteins (ORAI). In this process, STIM detects the depletion of Ca^2+^ in the ER and activates ORAI channels on the plasma membrane, allowing Ca^2+^ influx [52]. STIM and ORAI work together to signal the need to replenish intracellular Ca^2+^, which is essential for immune cell activation.

SOCE signaling is important for several T cell functions, such as T cell development and differentiation, stimulation of CD8^+^ T cells followed by the release of cytotoxic granules, cytokine production, and control of mast cell and neutrophil function [53]. In murine models of asthma, blocking CRAC channels effectively prevents Th2 cell-mediated responses [54], while mast cells from STIM1 or ORAI1 knockout mice exhibit impaired degranulation and reduced activation of transcription factors NF-AT and NF-κB [55,56]. In experimental models, such as allergic asthma in guinea pigs, the use of a STIM-ORAI coupling blocker—3-fluoropyridine-4-carboxylic acid (FPCA)—resulted in decreased airway hyperreactivity and inflammation [57]. Other studies using the STIM-ORAI antagonist SKF 96365 reduced key inflammatory cytokines, including IL-4, IL-5, IL-12, IL-13, IFN-γ, and TNF-α, and effectively reversed airway tissue remodeling in ovalbumin-induced asthma models [58].

Beyond immune cells, CRAC channels are also expressed in airway smooth muscle and epithelial cells, positioning them as attractive therapeutic targets for asthma treatment [59,60]. Studies have shown that the expression of STIM1 and ORAI1 proteins is positively modulated in the smooth muscle cells of tracheal and bronchial tissues in ovalbumin-challenged asthma models. Reducing the expression of these proteins inhibits smooth muscle cell chemotaxis and proliferation, underscoring the role of STIM1 and ORAI1 in smooth muscle remodeling in asthma [61].

### 3.2. Potassium Channels

Potassium (K^+^) channels play a crucial role in regulating immune cell function and airway smooth muscle tone, which are key factors in asthma pathophysiology. Calcium-activated potassium (K_Ca_) channels, including intermediate conductance (IK_Ca_) and large conductance (BK_Ca_), are particularly important in this context. These channels are involved in T cell activation and proliferation [62,63].

IK_Ca_ channels are expressed in various cell types found in the airways, including mast cells, macrophages, fibroblasts, T lymphocytes, epithelial cells, and smooth muscle cells [64,65]. IK_Ca_ channels can regulate key processes such as cell proliferation, chemotaxis, activation, and smooth muscle responsiveness, all of which contribute to the inflammatory and hyperreactive environment in asthmatic airways [66]. IK_Ca_ is also involved in mast cell degranulation and IgE-mediated histamine release [67]. A study has shown that the administration of an IK_Ca_ blocker was effective in reducing bronchoconstriction, pulmonary resistance, airway hyperresponsiveness to carbachol, and eosinophilia in bronchoalveolar fluid in an ovine model of mite-induced experimental asthma [66]. Similarly, the activation of BK_Ca_ channels with rottlerin has been found to attenuate airway hyperreactivity and cell infiltration in the lungs by lowering the production of Th2 cytokines in ovalbumin and mite-induced asthma models [68]. The involvement of IK_Ca_ and BK_Ca_ channels in regulating airway smooth muscle tone, bronchial hyperresponsiveness, and inflammation makes them promising targets for asthma treatment.

### 3.3. Chloride Channels

Chloride channels are widely expressed across various tissues and play essential roles in cell volume regulation, transepithelial transport, intracellular pH balance, and membrane excitability [69]. Of all chloride channels, the cystic fibrosis transmembrane conductance regulator (CFTR) is especially important. CFTR, a member of the ATP-binding cassette (ABC) transporter gene family, is unique in its ability to conduct chloride (Cl^−^) ions at high rates [70]. Located in the apical membrane of epithelial cells, CFTR facilitates the secretion of Cl^−^ through epithelial tissues, which influences the pH and mucus composition in the airways [71].

CFTR not only regulates Cl^−^ transport but also modulates other ion channels, such as epithelial sodium channels (ENaCs) [72], and interacts with calcium-activated chloride channels (CaCC) and outward rectifying chloride channels (ORCC) [73,74]. These interactions have significant implications for lung function and asthma pathophysiology. Research has shown that chronic exposure to Th2 cytokines, such as IL-4 and IL-13, increases CFTR activity [75,76], which has been demonstrated in both human airway tissues and mouse models of allergic asthma.

Furthermore, clinical studies [77] have suggested a potential link between CFTR dysfunction and asthma severity. In a multicenter study involving asthmatic patients, genetic polymorphisms in CFTR were more common in patients with severe asthma and hypersecretion. These findings suggest a correlation between CFTR mutations and poor clinical outcomes in asthma. The involvement of CFTR in immune regulation and its effects on epithelial ion transport make it a critical component in asthma, in cases associated with mucus hypersecretion and airway inflammation, although further research is needed to understand the relationship between CFTR and asthma pathogenesis fully.

### 3.4. TRP Channels

Transient receptor potential (TRP) channels play critical roles in physiological and pathological processes, including asthma. Among TRP channels, TRPA1, TRPV1, and TRPV4 are relevant to asthma due to their widespread expression in the respiratory system [78].

TRPA1 is found in sensory neurons, immune cells such as mast cells and T cells [79], and bronchial epithelial cells [80], where it plays a significant role in mediating the inflammatory response in respiratory conditions like asthma. Activation of TRPA1 by inhaled irritants leads to calcium influx and the release of inflammatory mediators, which contributes to the recruitment of neutrophils and eosinophils, exacerbating airway inflammation [81]. In mouse models, TRPA1 knockout mice (TRPA1^−/−^) demonstrated reduced immune cell infiltration in the bronchoalveolar fluid, further supporting the role of TRPA1 in asthma-related inflammation [82]. Similarly, TRPV1, expressed in airway epithelial cells and immune cells, has been linked to asthma severity. Studies have shown that patients with severe asthma exhibit elevated TRPV1 expression in the airway epithelium [83], and asthmatic children present higher levels of TRPV1 gene expression compared to healthy controls [84]. In animal models, TRPV1 knockout mice displayed reduced IgE levels and were protected from airway hyperresponsiveness, indicating the channel’s involvement in the allergic asthma phenotype [85]. TRPV4 plays a regulatory role in asthma. CFTR is expressed in airway smooth muscle, epithelial cells, and fibroblasts and plays a role in modulating airway remodeling. Studies in mice models have demonstrated that TRPV4 influences airway wall thickness, collagen synthesis, goblet cell recruitment, and fibrosis, which are key features of asthma pathology [86]. TRPV4 knockout could protect from airway remodeling in asthmatic mice model [87], emphasizing its role in asthma-related structural changes. Pertinent research indicates that obesity is a crucial factor in asthma [88], and the prevalence of obese asthmatic patients has risen markedly in recent years, with a significant proportion of these individuals experiencing more severe symptoms than those with typical asthma [89]. Dysfunction of TRP channels has been implicated in obesity [90]. More important, it has been found that TRPV4, TRPM8, and TRPC1 are primarily associated with obese asthma through their role in inflammation, and they are also linked to the production of fat and inflammatory factors [91]. Overall, TRP channels, including TRPA1, TRPV1, and TRPV4, contribute to asthma pathophysiology and the immune response by regulating inflammatory processes, immune cell recruitment, and airway remodeling, making them potential therapeutic targets for asthma management.

### 3.5. Proton Channels

The voltage-gated proton channel Hv1 plays a role in immune response and pH regulation. This channel is regulated by voltage [92,93] and has been identified in several cell types [94], including macrophages, blood cells, lung epithelial cells, skeletal muscle, and microglia. One of the primary functions of Hv1 channels is their involvement in maintaining pH homeostasis essential for cellular functions [95], including acid secretion in the airways [96,97]. In the lungs, Hv1 channels contribute to acid secretion into the alveolar lumen, and this increased acid secretion is thought to exacerbate inflammation during asthma.

Hv1 channels are important in the phagocytic system, a key component of the innate immune response [98]. Phagocytes, such as macrophages, neutrophils, and eosinophils, engulf and digest pathogens through a process called phagocytosis. During this process, phagocytes generate toxic reactive oxygen species (ROS) through an event known as the “respiratory burst,” which is essential for neutralizing and destroying engulfed pathogens. The enzyme NADPH oxidase is responsible for producing ROS. NADPH oxidase is an electrogenic enzyme, meaning it transports electrons across the phagocyte membrane, creating an electrical imbalance that may inhibit its activity. Hv1 channels play a critical role in maintaining charge balance during the respiratory burst by facilitating the movement of protons across the membrane. This proton movement compensates for the electron flow generated by NADPH oxidase, ensuring the continued production of ROS necessary for pathogen destruction. In this way, Hv1 channels are essential for the optimal function of NADPH oxidase-dependent ROS generation in phagocytic cells. In macrophages, Hv1 channels directly participate in the production of ROS during the respiratory burst [94], enabling effective phagocytosis and pathogen clearance. Similarly, eosinophils, which are key players in allergic asthma, rely on Hv1 channels for optimal ROS generation. Studies have shown that Hv1 is required in eosinophils not only for ROS production but also for preventing activation-induced cell death [99], which is crucial for sustaining immune response during inflammation.

In the context of asthma, deficiency of Hv1 channels has been linked to aggravated disease outcomes. For example, in mouse models of ovalbumin-induced allergic asthma, the absence of Hv1 channels has worsened lung inflammation [100], suggesting that Hv1 plays a protective role in regulating immune responses in asthma. This emphasizes the critical role of Hv1 channels in pathogen defense as well as in regulating the immune environment during inflammatory diseases such as asthma. However, excessive production of ROS may exacerbate airway inflammation and oxidative damage, leading to airway epithelial cell damage and airway remodeling. Increased ROS is strongly associated with oxidative stress in asthma and may promote airway constriction, mucus hypersecretion, and tissue fibrosis.

In summary, Hv1 channels play a key role in regulating oxidative stress in the immune system, particularly by maintaining charge balance during the phagocytic respiratory burst. Their involvement in ROS production by macrophages, neutrophils, and eosinophils highlights their importance in asthma pathogenesis. Understanding the role of Hv1 channels in these processes sheds light on new therapeutic avenues for managing asthma and its associated immune dysfunction [101].

### 3.6. Piezo Channel

Piezo-type mechanosensitive ion channel component 1 (PIEZO1) is a non-selective, mechanosensitive cation channel that functions as a mechanical transducer. It detects mechanical stimuli, such as pressure and stretch, in various cell types, including endothelial, epithelial, and immune cells [102]. PIEZO1 contributes to mechanotransduction, the process by which cells convert mechanical signals into biochemical responses.

In the lungs, PIEZO1 plays a significant role in regulating airway function. Activation of PIEZO1 through its specific agonist, Yoda1, has been shown to alter the biomechanics and contractile machinery of airway smooth muscle cells, leading to the relaxation of these cells [103]. This suggests that PIEZO1 may have therapeutic potential in modulating airway constriction, a key feature of asthma. Moreover, studies have found that PIEZO1 is highly expressed in the airway epithelium of asthmatic mice and have explored its role in regulating adherent junctions, which are crucial for maintaining the integrity of the airway epithelium’s protective barrier [104].

Recent discoveries have drawn significant attention to the role of Piezo channels in immune cell function [102]. In mechanosensitive organs like the lungs, Piezo1 activation in immune cells has been linked to the modulation of the inflammatory response. For instance, in lung macrophages and monocytes, Piezo1 activation has been shown to promote inflammation by enhancing the secretion of chemokines such as CXCL2 [105,106]. This pro-inflammatory signaling contributes to the recruitment of immune cells to the site of inflammation, exacerbating conditions like asthma, where airway inflammation is a central pathological feature. However, studies have demonstrated that PIEZO1 activation may negatively drive T-cell differentiation [107]. And in asthma, PIEZO1 also interacts with ILC2. Recent research has shown that Piezo1 channels can repress ILC2-driven type 2 inflammation and AHR [108]. This suggests that Piezo1 may act as an inhibitor effect in certain contexts, indicating a potential balancing role in immune regulation. By modulating the mechanical and immune environment of the lungs, PIEZO1 helps to fine-tune the inflammatory response, potentially offering new avenues for asthma treatment by targeting mechano-transduction pathways.

## 4. Future Directions and Challenges

Current research into ion channels in asthma has opened new frontiers in understanding how Ca^2+^, K^+^, Cl^−^, H^+^, TRP, and Piezo1 channels contribute to immune responses in the disease. These channels regulate key processes such as airway smooth muscle contraction, inflammatory mediator release, and immune cell activation, making them promising targets for therapeutic interventions. Although recent progress has been made in the functional characterization of the Ca^2+^, K^+^, and Cl^−^ channels in asthma (Figure 3), the role of Na^+^ channels, such as voltage-gated sodium channel (Na_v_), in this disease remains unknown. Given that voltage-gated sodium channels are highly expressed in immune cells in the lungs and play a significant role in airway defense, future studies are required to characterize the potential function of these channels in the pathogenesis of asthma.

Moreover, several existing treatment strategies already focus on modulating ion channels in asthma. For example, calcium channel blockers have shown efficacy in reducing bronchoconstriction, while potassium channel activators help manage airway hyperresponsiveness. Similarly, targeting TRP channels has been explored to reduce inflammation and oxidative stress in asthma. Despite these advances, there still are several challenges. One of the primary obstacles is the development of selective ion channel inhibitors that can target specific asthma phenotypes without causing systemic side effects. However, integrating ion channel modulation into personalized asthma treatment represents a significant opportunity for the future. Understanding how specific ion channel dysfunctions contribute to different asthma phenotypes could lead to more tailored therapies. This individualized approach could improve treatment outcomes by targeting each patienťs underlying disease mechanisms. The potential for ion channel-based therapies to revolutionize asthma treatment is significant, and ongoing research is needed to fully realize this opportunity.
